# *RHC* genotyping in Chinese Han population

**DOI:** 10.1186/s12864-024-10444-6

**Published:** 2024-05-28

**Authors:** Lin-Nan Shao, Zi-Wei Zheng, Shi-Hang Zhou, Wen-Qian Song, Yue-Xin Xia, Xiao-Hua Liang

**Affiliations:** 1 Dalian Blood Center, No.90 Yan’an Road, Zhongshan District, Dalian, 116001 China; 2 College of Medical Laboratory, Dalian Medical University, Dalian, 116044 Liaoning China

**Keywords:** RHC, RHc, Genotyping, SNP, Phenotype

## Abstract

**Background:**

The Rh blood group system is characterized by its complexity and polymorphism, encompassing 56 different antigens. Accurately predicting the presence of the C antigen using genotyping methods has been challenging. The objective of this study was to evaluate the accuracy of various genotyping methods for predicting the Rh C and to identify a suitable method for the Chinese Han population.

**Methods:**

In total, 317 donors, consisting 223 D+ (including 20 with the Del phenotype) and 94 D− were randomly selected. For *RHC* genotyping, 48C and 109bp insertion were detected on the Real-time PCR platform and −292 substitution was analyzed via restriction fragment length polymorphism (RFLP). Moreover, the promoter region of the *RHCE* gene was sequenced to search for other nucleotide substitutions between *RHC* and *RHc*. Agreement between prediction methods was evaluated using the Kappa statistic, and comparisons between methods were conducted via the χ^2^ test.

**Results:**

The analysis revealed that the 48C allele, 109bp insertion, a specific pattern observed in RFLP results, and wild-type alleles of seven single nucleotide polymorphisms (SNPs) were in strong agreement with the Rh C, with Kappa coefficients exceeding 0.8. However, there were instances of false positives or false negatives (0.6% false negative rate for 109bp insertion and 5.4-8.2% false positive rates for other methods). The 109bp insertion method exhibited the highest accuracy in predicting the Rh C, at 99.4%, compared to other methods (*P* values≤0.001). Although no statistical differences were found among other methods for predicting Rh C (*P* values>0.05), the accuracies in descending order were 48C (94.6%) > rs586178 (92.7%) > rs4649082, rs2375313, rs2281179, rs2072933, rs2072932, and RFLP (92.4%) > rs2072931 (91.8%).

**Conclusions:**

None of the methods examined can independently and accurately predict the Rh C. However, the 109bp insertion test demonstrated the highest accuracy for predicting the Rh C in the Chinese Han population. Utilizing the 109bp insertion test in combination with other methods may enhance the accuracy of Rh C prediction.

**Supplementary Information:**

The online version contains supplementary material available at 10.1186/s12864-024-10444-6.

## Introduction

The Rh blood group system is a complex, polymorphic system consisting of 56 antigens [1], with Rh DCcEe being the most clinically relevant in both transfusion and obstetric medicine [2]. These antigens are encoded by two highly similar *RH* genes, namely *RHD* and *RHCE*, sharing 93.8% homology across introns and coding exons [3]. In addition to the anti-D antibody, alloantibodies produced against Rh CcEe antigens are known to be involved in hemolytic disease of the fetus and newborn as well as hemolytic transfusion reactions [4]. Eighty-nine retrospective analyses have demonstrated that the average prevalence of alloantibodies in Chinese population was 0.34%, and the most frequent alloantibodies are those targeting the Rh system (71.7%), including anti-E (33.9%), anti-c (10.9%), anti-C (8.1%), and anti-e (4.8%) [5].

Early knowledge of the fetal Rh blood group type is important for predicting and managing the clinical course. However, invasive methods such as fetal blood sampling or amniocentesis can pose a significant risk to the fetus and, thus, are unsuitable for routine testing [6]. To mitigate these risks, determining the fetal genotype through circulating cell-free fetal DNA in maternal plasma seems to be the most promising approach [7, 8]. Transfusion-related complications are common in patients with sickle cell disease (SCD) and thalassemia owing to the high rates of alloimmunization [9]. The prevalence of alloimmunization ranges from 18% to 75% in SCD and from 4% to 37% in thalassemia [10, 11]. Consequently, many transfusion services have implemented prophylactic phenotype matching for Rh CcEe antigens. However, if these patients have been typed only for ABO and D before their first transfusion, subsequent serological typing of Rh CcEe antigens may lead to mixed-field agglutination due to multiple transfusions [12]. Thus, it is important to determine the *RHC*/*c* and *RHE*/*e* genotypes in these cases.

The *RHCE* gene is responsible for encoding the Rh E/e and Rh C/c antigens [13]. The Rh E antigen differs from the Rh e antigen by a single amino acid substitution of proline to alanine at position 226 (676C>G) in exon 5 [14]. The molecular basis for the Rh C/c polymorphism is more intricate, typically involving six nucleotide substitutions within the RHCE gene, which result in four amino acid changes (C/c): Cys16Trp (48C/G) encoded by exon 1, Ile60Leu (178A/C), Ser68Asn (203G/A), and Ser103Pro (307T/C) encoded by exon 2 [15, 16]. Among these substitutions, only the Ser103Pro substitution is anticipated to be located extracellularly, specifically in the second extracellular loop [15]. The Rh c antigen is almost entirely determined by the presence of the 307C nucleotide [17]. Several *RHC* identification methods have been established. Faas et al. [18] identified *RHC* based on the nt48 polymorphism, whereas Avent [19] identified *RHC* based on the 109bp insertion in intron 2. Although both these methods demonstrate high accuracy, there have been reports of false positive or false negative results when these methods were used separately [20, 21].

A promoter is a sequence located upstream of the 5′-flanking region of a gene, and alterations in this region can influence gene expression and regulation. Tanaka et al. conducted a study using a restriction fragment length polymorphism (RFLP) assay for *RHC* genotyping based on the nucleotide differences at position −292 in the promoter region of *RHCE* [22]. Nevertheless, similar to the 48C approach, this RFLP assay also yielded false positive outcomes. The existence of additional nucleotide substitutions within *RHCE* promoter, and whether they can accurately forecast the Rh C, remains unresolved.

In the current study, we assessed various methodologies for *RHC/c* genotyping among the Chinese Han population, including those based on the 48C method, the 109bp insertion approach, the 307C method, and RFLP analysis of the −292 substitution. Additionally, we sequenced the promoter region of the *RHCE* gene to detect any further nucleotide substitutions distinguishing the *RHC* and *RHc* alleles. The objective of this research was to evaluate the accuracy of diverse genotyping techniques for predicting the Rh C and to identify a method that is appropriate for the Chinese Han population.

## Materials and methods

### Patients and samples

The study participants were limited to healthy unrelated Chinese Han blood donors. In total, 317 donors were randomly selected. All participants provided written informed consent to undergo sequence analysis. All procedures were performed in accordance with the ethical standards of the responsible committee on human experimentation (institutional and national). The study was conducted according to the guidelines of the Declaration of Helsinki, and approved by the Dalian Blood Center Ethics Committee (protocol code: No.2 and date of approval: 2022.2.21).

### Serological typing of Rh blood group

Rh DCcEe phenotyping was conducted using standard monoclonal antisera (Shanghai Hemo-Pharmaceutical & Biological Co., Ltd., Shanghai, China) via tube agglutination tests. Samples typed as D− for the Del phenotype through an adsorption-elution test in tubes. If the result was positive, the sample was determined to belong to the Del phenotype, otherwise it was determined to be truly D− phenotype.

### DNA extraction

Genomic DNA was extracted from peripheral whole blood samples treated with proteinase K. The extraction was performed using a commercially available DNA isolation kit on the MagCore Automated Nucleic Acid Extractor (RBC Bioscience) according to the manufacturer’s instructions.

### Real-time PCR

The Rh C-associated 48C and 109bp insertion, as well as the Rh c epitope-specifying 307C were genotyped base on real-time PCR platform as previously described [23].The primer and probe sets used for each genotyping assay are listed in Table 1. To avoid false-negative results, an internal control PCR was included in the analysis. This was achieved by adding a glyceraldehyde-3-phosphate dehydrogenase (GAPDH) primer and probe set to the PCR mixture.

**Table 1:** Primers and probes

Primer/probe		Sequence
Real-time PCR	RHc307C_F	5’-TGGGCTTCCTCACCTCAAA-3’
	RHc307C_R	5’-TGATGACCACCTTCCCAGG-3’
	RHc307_Probe	5’-(FAM)CAATCCTGCTGGACGGCTTCCTGA(BHQ1)-3’
	RHC109bp_F	5’-CATTGCTATAGCTTAAGGACTCA-3’
	RHC109bp_R	5’-ATGATTGTACCACTGGGAAG-3’
	RHC109bp_Probe	5’-(FAM)CAACACCAAACCAGGGCCACC(BHQ1)-3’
	RHC48C_F	5’-CTGCCTGCCCCTCTGC-3’
	RHC48C_R	5’-CTTGATAGGATGCCACGAGCC-3’
	RHC48_Probe	5’-(JOE)ACCCACTATGACGCTTCCTTAGAGGAT(BHQ1)-3’
	GAPDH_F	5’-CCCCACACACATGCACTTACC-3’
	GAPDH_R	5’-CCTAGTCCCAGGGCTTTGATT-3’
	GAPDH_Probe	5’-(TAMRA)AAAGAGCTAGGAAGGACAGGCAACTTGGC(BHQ2)-3’
RFLP	RFLP_F	5’-TCCACCTTCCACTTCCCTGT-3’
	RFLP_R	5’-AGAGGGCATTCTATTCCTTTGA-3’
	RFLP_F'	5’-(FAM)TCCACCTTCCACTTCCCTGT-3’
	RFLP_R'	5’-(FAM)AGAGGGCATTCTATTCCTTTGA-3’
Sequencing	RHCE_F	5’-TCCACCTTCCACTTCCCTGT-3’
	RHCE_R	5’-CTTGATAGGATGCCACGAGCC-3’
	RHD_F	5’-TCCACTTTCCACCTCCCTGC-3’
	RHD_R	5’-CTTGATAGGATGCCACGAGCC-3’

*RFLP* Restriction fragment length polymorphism

### RFLP

The genotyping of RFLP was performed using the forward and reverse primer pair RFLP-F and RFLP-R (Table 1). The final reaction volume for the PCR mixtures was 25 μL. The mixtures included 1 μL of purified genomic DNA (approximately 50–200 ng), 1 μL of forward and reverse primers (20 μM), 12.5 μL of AmpliTaq Gold 360 Master Mix (Applied Biosystems), 2 μL of 360 GC enhancer, and 7.5 μL of distilled water. The PCR procedure was as follows: 95°C for 10 min, followed by 35 cycles of 95°C for 30 s, 63°C for 30 s, and 72°C for 2 min, and a final extension step at 72°C for 7 min. The PCR products were digested with the enzyme FlashCut *Ssp*I (Monad Biotech Co., Ltd., Shanghai, China) and analyzed by 3% agarose gel electrophoresis (Shanghai Baygene Biotechnology Co., Ltd., Shanghai, China).

### Sequencing

The promoter region and exon 1 of *RHCE* and *RHD* were amplified using PCR. *RHCE*- and *RHD-*specific primers are shown in Table 1. PCR amplification was performed as previously described [24].The amplification products were 1290 bp (spanning from −1142 to +148) for *RHCE* and 1286 bp (spanning from −1138 to +148) for *RHD*. The amplified products were then subjected to direct sequencing using forward and reverse primers on an ABI 3730 XL DNA analyzer (Applied Biosystems), according to the manufacturer’s instructions. The sequencing results were analyzed using DNAstar software.

### Statistical analysis

Statistical analysis to assess agreement was conducted using the Kappa statistic. Interpretation of Kappa coefficients is as follows: a coefficient between 0.00–0.20, 0.21–0.40, 0.41–0.60, 0.61–0.80 and 0.81–1.00 respectively corresponded to slight, fair, moderate, substantial and almost perfect agreement [25]. Comparisons between different methods were assessed using χ^2^ test. The *P* value of less than 0.05 was considered statistically significant difference. The statistical analyses were performed in SPSS software (version 21.0). PASS v.11 was used for calculating power analysis. The power was estimated more than 0.9 along with 317 participants with a two-sided significance level of 0.05 was to be achieved.

## Results

### Serology

Among the 317 donors included in this study, the cohort comprised 223 Rh D+ individuals (20 of whom had the Del phenotype) and 94 Rh D− individuals. Additionally, eight different Rh CcEe serotypes were identified (Table 2).

**Table 2:** Genotyping for* RHC* and *RHc*

Serotype		Real-time PCR					RFLP		
		48C	109bp	307C	n	%	Fragment	n	%
D+	ccee	N	DEL	P	3	0.9	2	3	0.9
	ccEe	N	DEL	P	9	2.8	2	9	2.8
	ccEe	P^a^	DEL	P	2	0.6	3^a^	2	0.6
	ccEE	N	DEL	P	16	5.0	2	15	4.7
	ccEE						3^a^	1	0.3
	Ccee	P	INS	P	20	6.3	1^a^	3	0.9
	Ccee	P	DEL^b^	P	1	0.3	3	18	5.7
	CcEe	P	INS	P	59	18.6	3	60	18.9
	CcEe	P	DEL^b^	P	1	0.3			
	CcEE	P	INS	P	1	0.3	3	1	0.3
	CCee	P	INS	N	89	28.1	1	89	28.1
	CCEe	P	INS	N	2	0.6	1	2	0.6
Del	Ccee	P	INS	P	16	5.0	3	14	4.4
	Ccee						1^a^	2	0.6
	CcEe	P	INS	P	2	0.6	3	2	0.6
	CCee	P	INS	N	2	0.6	1	2	0.6
D-	ccee	N	DEL	P	53	16.7	2	53	16.7
	ccee	P^a^	DEL	P	14	4.4	3^a^	12	3.8
	ccee						1^a^	2	0.6
	ccEe	N	DEL	P	7	2.2	2	7	2.2
	ccEe	P^a^	DEL	P	1	0.3	3^a^	1	0.3
	Ccee	P	INS	P	14	4.4	3	14	4.4
	CcEe	P	INS	P	1	0.3	1^a^	1	0.3
	CCee	P	INS	N	4	1.3	1	4	1.3

^a^false positive for RhC

^b^false negative for RhC; *Del* RhD-elute, *D-* RhD-negative, *D+* RhD-positive. *n* number. *N* Negative; *P* Positive, *DEL* Deletion, *INS* Insertion, *RFLP* Restriction fragment length polymorphism

### Real-time RCR

**Fig. 1:**
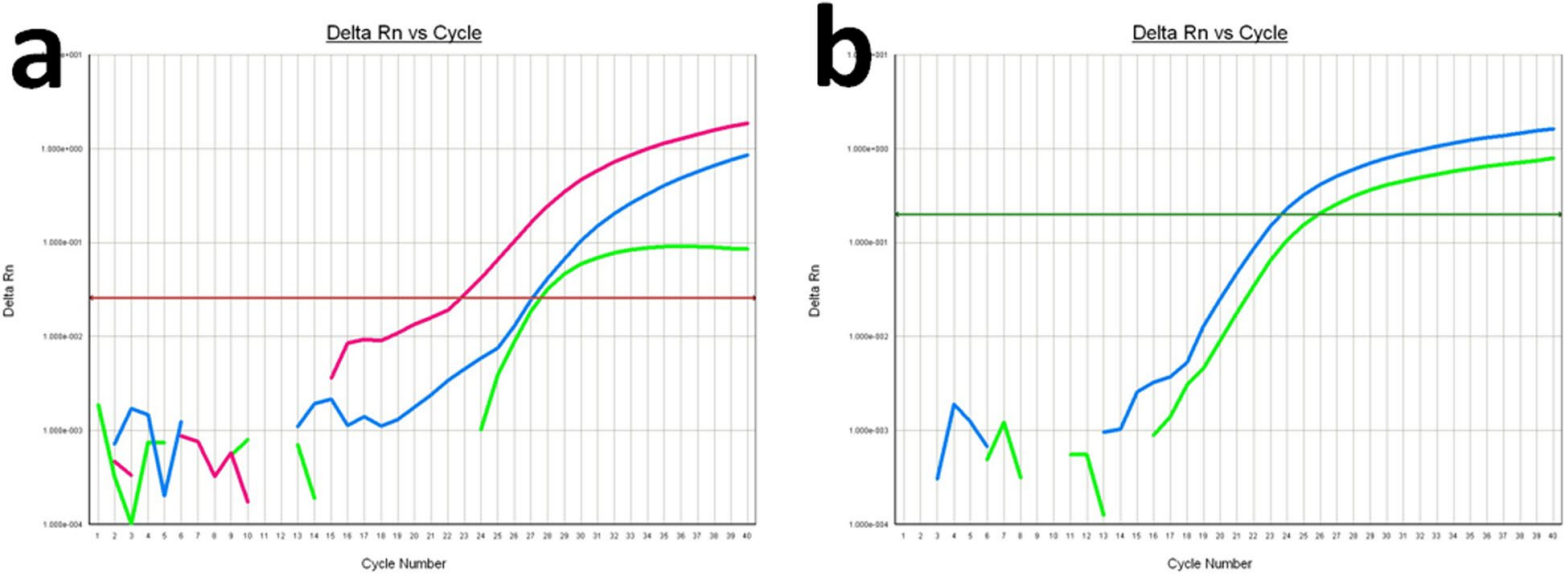
Real-time PCR amplification curve for an individual with Rh Cc antigen. **a** Real-time PCR logarithmic plot for Rh C-associated 48C, 109bp insertion, and internal control: The amplification curves for Rh C-associated 48C positive (pink curve), 109bp insertion (blue curve), and internal control (green curve) are shown. **b** Real-time PCR logarithmic plot for Rh c epitope-specifying 307C and internal control: The amplification curves for Rh c epitope-specifying 307C positive (blue curve) and internal control (green curve) are shown. The y-axis represents the relative fluorescence units (Delta Rn) of the reporter dye during the final cycles, and the x-axis represents the cycle number

A total of 317 donors were genotyped for *RHC*/*c* base on real-time PCR, targeting Rh C-associated 48C and 109bp insertion, and Rh c epitope-specifying 307C. Figure 1 displays the real-time PCR amplification curve of an Rh Cc individual. The genotyping results are listed in Table 2. These results were compared with the serotyping results to assess the correlation between the different genotyping methods. A strong agreement was observed between the presence of the 48C allele and the Rh C antigen (94.6%, 300/317; Kappa=0.874, *P*<0.001), yet only a few false positive results were identified in individuals who were Rh cc by serological typing but positive for the 48C allele by genotyping (5.4%, 17/317). The 109bp insertion genotyping result for Rh C was more reliable than that for the 48C (*P*=0.001), with only two false negatives (0.6%, 2/317; Kappa=0.986, *P*<0.001) found in Rh CcD+ individuals. The genotyping results using the 307C allele for *RHc* showed complete concordance with the serotyping results, without any false negative or false positive findings.

### RFLP

**Fig. 2:**
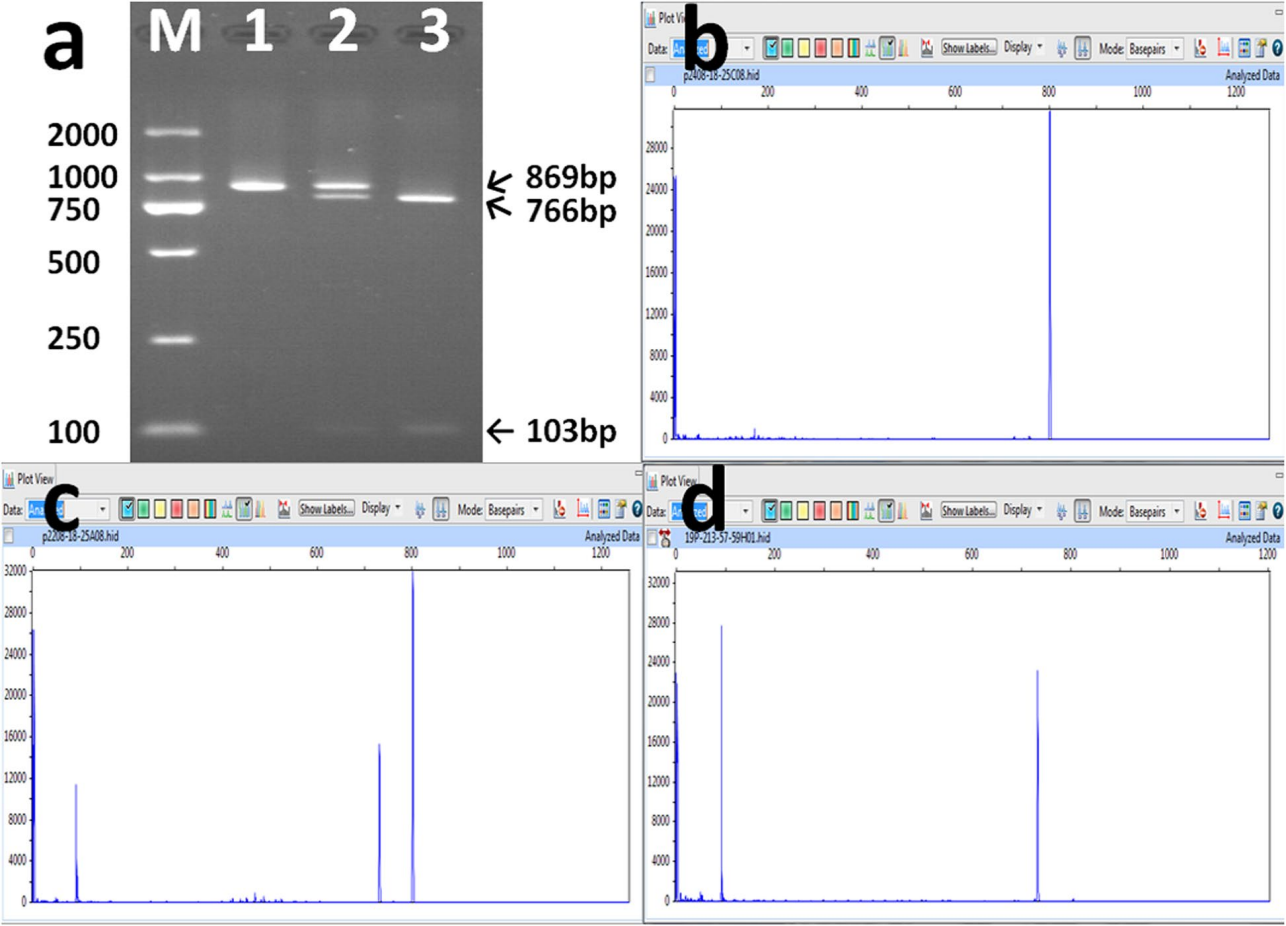
PCR-RFLP genotyping for *RHC/c*. **a** Cropped gel electrophoresis results are shown. Lane 1, Rh CC phenotype; Lane 2, Rh Cc phenotype; Lane 3, Rh cc phenotype. Original gels are presented in Supplementary Figure 1. **b** Capillary electrophoresis results for an individual with Rh CC genotype. A single peak is observed. **c** Capillary electrophoresis result for an individual with Rh Cc genotype. Three peaks are observed. **d** Capillary electrophoresis results for an individual with Rh cc genotype. Two peaks are observed

Amplification products of 869 bp were obtained. As the *Ssp*I digestion site is specific to *RHc* and not *RHC*, the products of *RHc* were expected to be digested into 766-bp and 103-bp fragments. The results of gel electrophoresis are shown in Fig. 2a. Original gels are presented in Supplementary Figure 1. Owing to the short length of the 103-bp fragment, it was difficult to observe it clearly on gel electrophoresis. Therefore, we improved this method to enhance the visualization and accuracy of the results. To overcome the limitations of gel electrophoresis, an improved method was implemented using primers RFLP_F′ and RFLP_R′ labeled with the fluorescent dye FAM at their 5′ ends (Table 1) to replace RFLP_F and RFLP_R. The digested products were detected using capillary electrophoresis on an ABI 3730 XL DNA analyzer. The capillary electrophoresis results are shown in Fig. 2b, c, and d. Restriction enzyme cutting site is shown in Fig, 3. The RFLP genotyping results of *RHC/c* are listed in Table 2. The specific pattern observed in the capillary electrophoresis results showed a high concordance with the serological typing results (92.4%, 293/317, Kappa=0.877, *P*<0.001). However, a few false positive results for Rh C were noted (7.6%, 24/317) in individuals identified as Rh Cc and Rh cc by serological typing. The RFLP result for Rh C was relatively less reliable compared to the genotyping result for the 109bp insertion (*P<*0.001), but no statistically significant difference was observed when compared to the 48C genotyping results.

**Fig. 3:**
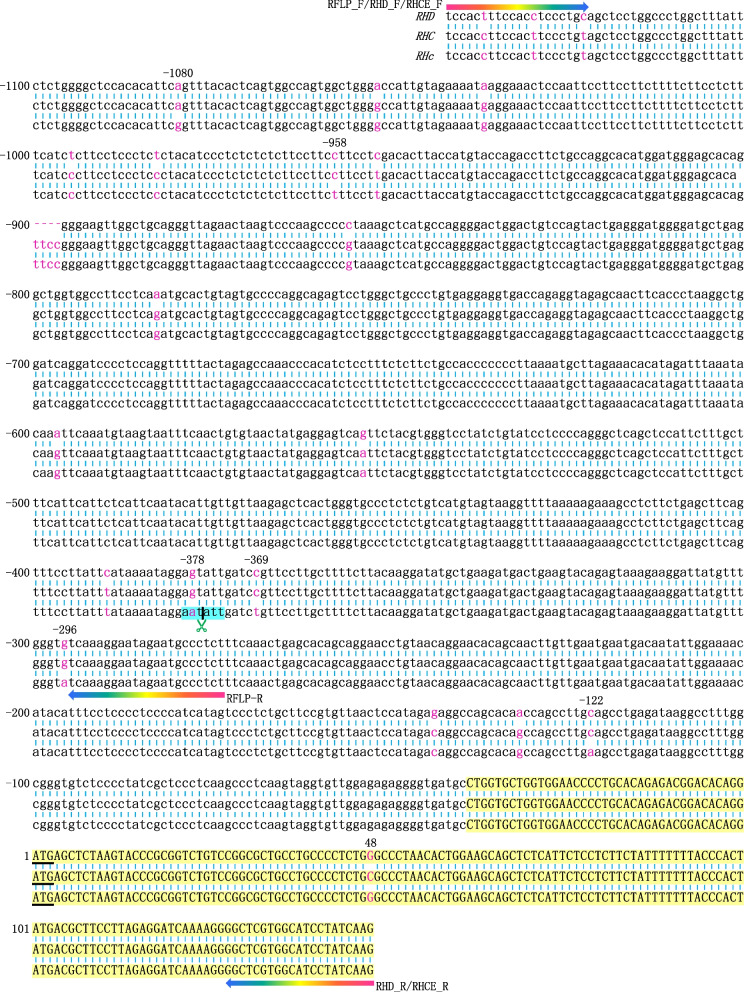
Nucleotide sequences of the 5′ flanking regions of *RHD* and *RHC/c*. The restriction enzyme cutting site is highlighted in blue. The primers are indicated by iridescent arrows. The nucleotides of exon 1 are highlighted in yellow with uppercase letters, and the initiation codon ATG is underlined. The positions with nucleotide differences are marked in red font

### Sequencing

The promoter and exon 1 of the *RHCE* were sequenced, revealing seven SNPs: rs4649082 (−1080A/G), rs2375313 (−958C/T), rs2281179 (−378G/A, defined as −292 in literature [22]), rs2072933 (−369C/T), rs2072932 (−296G/A), rs2072931 (−122C/A), and rs586178 (48C/G), which corresponding to Rh C/c (Fig. 3). The genotype distributions of these SNPs are presented in Table 3. The wild-type alleles of these SNPs were associated with the Rh C. When compared to serotyping results, the false positive rates were 7.3% (23/317, Kappa=0.891, *P*<0.001) for rs586178; 7.6% (24/317, Kappa=0.886, *P*<0.001) for rs4649082, rs2375313, rs2281179, rs2072933, and rs2072932; and 8.2% (26/317, Kappa=0.887, *P*<0.001) for rs2072931. No false negative results observed. Moreover, *RHD* promoter deletions were detected in 81 of the 94 Rh D− individuals, and sequencing revealed no mutations in the *RHD* promoter region associated with the Rh C/c phenotype. The SNPs for Rh C were also found to be less reliable compared to the 109bp insertion genotyping result (*P<*0.001). Using different methods, discrepancies were noted in Rh C prediction among individuals with the same phenotype or within the same individual, leading to slightly varying accuracies. Although no statistical difference was found by the χ^2^ tests among these methods (*P* values>0.05), the accuracies were ranked as follows: 48C (94.6%) > rs586178 (92.7%) > rs4649082, rs2375313, rs2281179, rs2072933, rs2072932 and RFLP (92.4%) > rs2072931 (91.8%). Furthermore, the proportion of false positives in Rh D− individuals was higher than that in Rh D+ individuals (*P* values<0.001). This discrepancy may be attributed to the differing proportions of the *c* allele between Rh D+ and Rh D− individuals (35.9% vs. 87.8%), with the ccee phenotype being dominant in Rh D− individuals.

**Table 3:** Sequencing in the promoter and exon 1 of the *RHCE*

Serotype		Sequencing								
		rs4649082	rs2375313	rs2281179	rs2072933	rs2072932	rs2072931	rs586178	n	%
		−1080A/G	−958C/T	−378G/A	−369C/T	−296G/A	−122C/A	48C/G		
D+	ccee	GG	TT	AA	TT	AA	AA	GG	3	0.9
	ccEe	GG	TT	AA	TT	AA	AA	GG	9	2.8
	ccEe	AG^a^	CT^a^	AG^a^	CT^a^	AG^a^	AC^a^	CG^a^	2	0.6
	ccEE	GG	TT	AA	TT	AA	AA	GG	14	4.4
	ccEE	GG	TT	AA	TT	AA	AC*	GG	1	0.3
	ccEE	AG^a^	CT^a^	AG^a^	CT^a^	AG^a^	AC^a^	GG	1	0.3
	Ccee	AG	CT	AG	CT	AG	AC	CG	18	5.7
	Ccee	AA^a^	CC^a^	GG^a^	CC^a^	GG^a^	CC^a^	CC^a^	3	0.9
	CcEe	AG	CT	AG	CT	AG	AC	CG	59	18.6
	CcEe	AG	CT	AG	CT	AG	CC^a^	CG	1	0.3
	CcEE	AG	CT	AG	CT	AG	AC	CG	1	0.3
	CCee	AA	CC	GG	CC	GG	CC	CC	89	28.1
	CCEe	AA	CC	GG	CC	GG	CC	CC	2	0.6
Del	Ccee	AG	CT	AG	CT	AG	AC	CG	14	4.4
	Ccee	AA^a^	CC*^a^	GG^a^	CC^a^	GG^a^	CC^a^	CC^a^	2	0.6
	CcEe	AG	CT	AG	CT	AG	AC	CG	2	0.6
	CCee	AA	CC	GG	CC	GG	CC	CC	2	0.6
D-	ccee	GG	TT	AA	TT	AA	AA	GG	53	16.7
	ccee	AG^a^	CT^a^	AG^a^	CT^a^	AG^a^	AC^a^	CG^a^	12	3.8
	ccee	AA^a^	CC^a^	GG^a^	CC^a^	GG^a^	CC^a^	CC^a^	2	0.6
	ccEe	GG	TT	AA	TT	AA	AA	GG	7	2.2
	ccEe	AG^a^	CT^a^	AG^a^	CT^a^	AG^a^	AC^a^	CG^a^	1	0.3
	Ccee	AG	CT	AG	CT	AG	AC	CG	14	4.4
	CcEe	AA^a^	CC^a^	GG^a^	CC^a^	GG^a^	CC^a^	CC^a^	1	0.3
	CCee	AA	CC	GG	CC	GG	CC	CC	4	1.3

^a^false positive for RhC; *Del* RhD-elute, *D-* RhD-negative, *D+* RhD-positive, *n* number

## Discussion

The molecular genetics of blood groups, particularly the *RH* system, has become increasingly important in transfusion medicine. Numerous methods have been developed to predict the Rh C across diverse ethnic groups. However, false positive or false negative predictions of the Rh C phenotype continue to occur. Variations in the genetic sequences of the *RH* genes among different ethnic groups necessitate the investigation of more appropriate methods for predicting the Rh C within specific populations, such as the Chinese Han population. Our study revealed a strong association between the 48C allele, 109bp insertion, specific RFLP patterns, and wild-type alleles of seven SNPs with the Rh C antigen, as indicated by Kappa coefficients greater than 0.8. Nonetheless, some false positive or false negative results were encountered (0.6% false negative rate for the 109bp insertion and 5.4-8.2% false positive rates for other methods). The 109bp insertion method exhibited the highest accuracy (99.4%) in predicting the Rh C, compared to other methods (*P* values ≤ 0.001).

*RHC* genotyping revealed two kinds of discrepancies. First, false positive results were encountered in the 48C, RFLP and sequencing methods. Prior studies have also documented false positive results in various populations [21]. Tanaka et al. reported that the polymorphism at the -292 position in the promoter region of *RHCE* could predict the Rh C phenotype using RFLP, with a low false positive rate (2.6%), and these results were exclusively observed in individuals with the Rh ccD− phenotype [22]. This is at odds with our findings, which demonstrated false positive results in both Rh D+ and Rh D− individuals, as well as in those with Rh cc and Rh CC phenotypes, within the Chinese Han population. This discrepancy may be attributed to ethnic variations and the diverse genetic backgrounds of individuals. Second, the 109bp insertion method yielded a false negative result in two Rh CcD+ Chinese Han donors (0.6%, 2/317) in this study. These two donors did not exhibit false positives in the 48C and −292 RFLP genotyping. Tax et al. investigated multiple ethnic groups and also observed false negative results with the 109bp insertion in intron 2, particularly in white and black individuals, with a higher proportion observed in black individuals [20]. This may be attributed to the presence of the r’s haplotype, which comprises a hybrid *RHD-CE-D* gene that lacks the *RHCE* intron 2 sequences [18]. This indicates that a method optimal for the Chinese Han population may not be appropriate for white and black individuals.

Among the positions examined, only the 307C variant, unique to the *RHc* gene and absent in *RHD*, was found to be strongly associated with the Rh c. Notably, the 307T allele is necessary but not sufficient for Rh C specificity, due to the identity of exon 2 in both *RHD* and *RHC*, which code for Ser103 [17]. Faas et al. reported that a particular mutation, 307T>C, in *RHD* can lead to Rh c expression even in the absence of an *RHc* allele. This indicates that the 307C site is critical for the manifestation of the Rh c phenotype [26].

To the best of our knowledge, this study represents the first to evaluate prediction methods for Rh C and to investigate polymorphisms in the *RHCE* promoter region, aiming to identify nucleotide substitutions between *RHC* and *RHc* alleles within the Chinese Han population. We identified five previously unstudied specific SNPs in the *RHCE* promoter regions capable of predicting the Rh C, although these SNPs may also yield false positive results. This comparative analysis employs the data obtained to inform decisions regarding the most reliable molecular approach for Rh C prediction. More accurate Rh C prediction results can be obtained in cases where direct phenotyping is not possible. Moreover, the study enhances the database of *RHCE* promoter region polymorphisms in the Chinese Han population, offering a molecular foundation for future research. A limitation of this work is the insufficient length of the explored *RHCE* promoter region, which may prevent finding of accurate Rh C prediction sites. Future research will involve expanding the sample size and employing more advanced technologies, such as third-generation sequencing, to examine the upstream, downstream, and intronic regions of the *RHCE* gene within the Chinese Han population, thereby uncovering additional SNPs that could predict Rh C with high accuracy.

In conclusion, the methods mentioned above alone cannot independently and accurately predict Rh C. The 109bp insertion test showed the highest level of accuracy for predicting the Rh C in the Chinese Han population. The 109bp insertion test combining with other methods can improve the accuracy of predicting Rh C.

### Supplementary Information

Supplementary Material 1. 

## Data Availability

The datasets generated and/or analysed during the current study are available in the Zenodo repository (10.5281/zenodo.11192240).
