# Supplementary figures and images for: RHC genotyping in Chinese Han population
Source: BMC Genomics. 2024 May 28;25:525. doi: 10.1186/s12864-024-10444-6 (PMC11134930; doi:10.1186/s12864-024-10444-6)

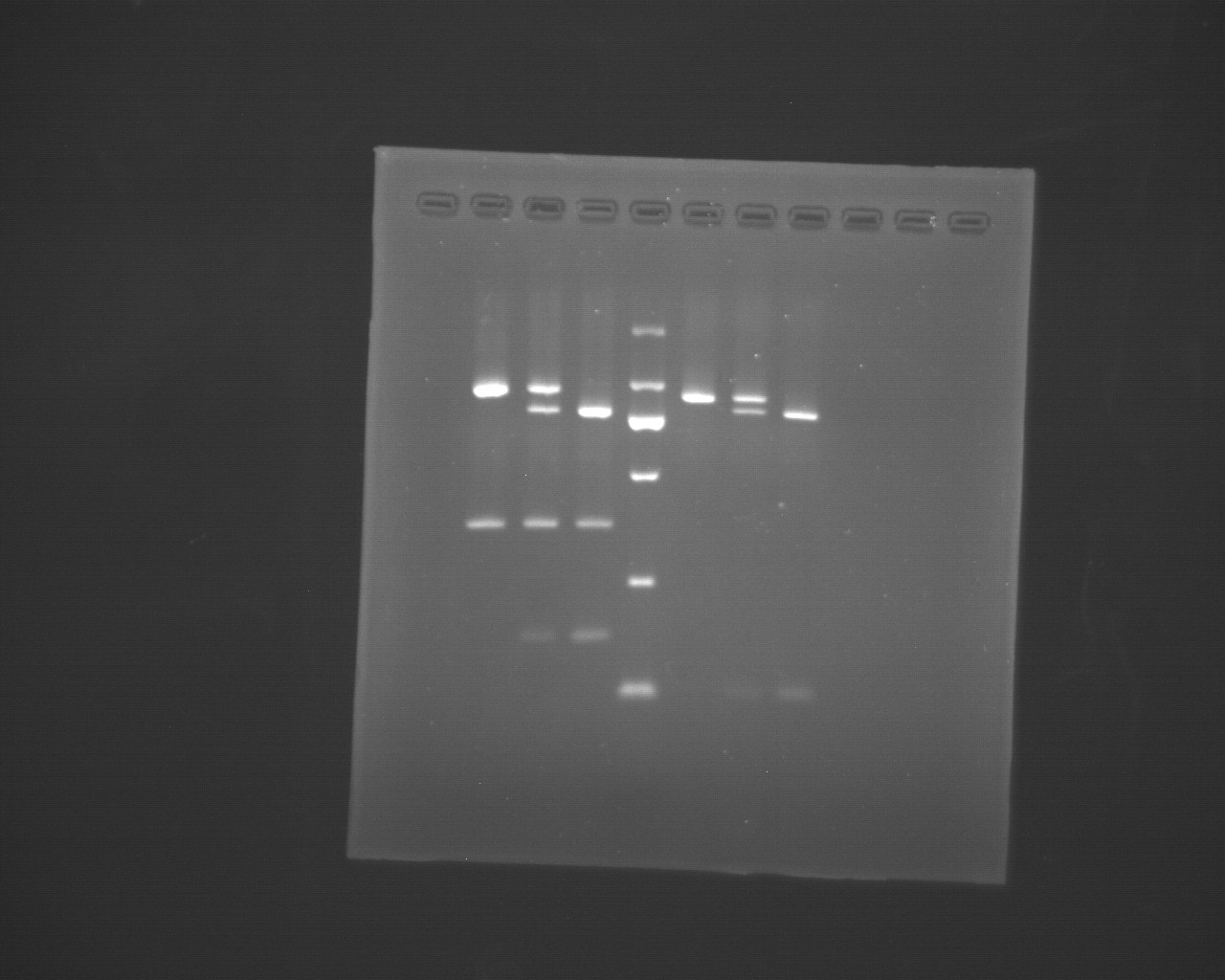

Supplement: Supplementary file 1 — Supplementary Material 1. [file 12864_2024_10444_MOESM1_ESM.jpg]
